# Effect of Human Mesenchymal Stem Cells on Xenogeneic T and B Cells Isolated from Lupus-Prone MRL*.Fas*^lpr^ Mice

**DOI:** 10.1155/2020/5617192

**Published:** 2020-03-05

**Authors:** Hong Kyung Lee, Eun Young Kim, Hyung Sook Kim, Eun Jae Park, Hye Jin Lee, Tae Yong Lee, Kyung Suk Kim, Sang-Cheol Bae, Jin Tae Hong, Youngsoo Kim, Sang-Bae Han

**Affiliations:** ^1^College of Pharmacy, Chungbuk National University, Cheongju, Chungbuk 28160, Republic of Korea; ^2^Bioengineering Institute, Corestem Inc., Seoul 04763, Republic of Korea; ^3^Hanyang University Hospital for Rheumatic Diseases, Seoul 04763, Republic of Korea

## Abstract

Systemic lupus erythematosus (SLE) is an autoimmune disease, which is characterized by hyperactivation of T and B cells. Human mesenchymal stem cells (hMSCs) ameliorate the progression of SLE in preclinical studies using lupus-prone MRL*.Fas*^lpr^ mice. However, whether hMSCs inhibit the functions of xenogeneic mouse T and B cells is not clear. To address this issue, we examined the *in vitro* effects of hMSCs on T and B cells isolated from MRL*.Fas*^lpr^ mice. Naïve hMSCs inhibited the functions of T cells but not B cells. hMSCs preconditioned with IFN-*γ* (i) inhibited the proliferation of and IgM production by B cells, (ii) attracted B cells for cell–cell interactions in a CXCL10-dependent manner, and (iii) inhibited B cells by producing indoleamine 2,3-dioxygenase. In summary, our data demonstrate that hMSCs exert therapeutic activity in mice in three steps: first, naïve hMSCs inhibit the functions of T cells, hMSCs are then activated by IFN-*γ*, and finally, they inhibit B cells.

## 1. Introduction

Mesenchymal stem cells (MSCs) are multipotent progenitor cells and have broad immunoregulatory properties on T cells (including Tregs), B cells, and dendritic cells [1]. MSCs are well known to inhibit T cell functions by producing soluble factors, such as tumor growth factor- (TGF-) *β*, prostaglandin E_2_ (PGE_2_), and indoleamine 2,3-dioxygenase (IDO), and to induce T cell apoptosis by expressing FasL and PD-L1 for contact-dependent inhibition [2, 3]. However, the effect of MSCs on B cells is controversial: MSCs inhibited proliferation, antibody production, and migration of B cells in some studies [4–6], but had no effect or even increased these functions in others [7–11]. It was also reported that MSCs indirectly inhibit B cell proliferation by suppressing helper T cell functions [7, 12]. Generally, the inhibitory mechanisms of MSCs have been studied *in vitro* by using species-matched autologous and allogeneic target immune cells, but not species-mismatched xenogeneic immune cells.

Systemic lupus erythematosus (SLE) is characterized by the production of autoantibodies to ubiquitous self-antigens [13]. In preclinical studies, the transfer of human MSCs (hMSCs) to lupus-prone MRL/MpJ-*Fas^lpr^* (called MRL.*Fas*^lpr^ hereafter) mice increased their survival and decreased the anti-dsDNA antibody level and nephritis [14]. However, it is still unclear whether hMSCs inhibit mouse T and B cells in this xenogeneic animal model. Here, we addressed this issue and investigated the interaction between hMSCs and mouse B cells in detail, since B cells play a critical role in SLE pathogenesis [13]. We found that naïve hMSCs inhibited mouse T cells only, whereas priming of hMSCs with IFN-*γ* rendered them capable of inhibiting mouse B cells in a CXCL10- and IDO-dependent manner.

## 2. Materials and Methods

### 2.1. Mesenchymal Stem Cells

Human bone marrow-derived MSCs were obtained from Corestem Inc. (Seoul, Korea) [15]. In brief, bone marrow was aspirated from the posterior iliac crest of healthy donors and mononuclear cells were collected by density gradient centrifugation. These cells were cultured in CSMB-A06 medium (Corestem Inc.) containing 10% fetal bovine serum (BD Biosciences, Franklin Lakes, NJ, USA), 2.5 mM l-glutamine, and penicillin/streptomycin (WelGene, Gyeonggi, Korea) in a 5% CO_2_ incubator at 37°C for 3–5 passages. After washing out nonadherent cells, the adherent cells retained the canonical phenotype of MSCs (CD29^+^ CD44^+^ CD73^+^ CD105^+^ CD90^+^ CD34^−^ CD45^−^ HLA-DR^−^) and were used in the experiments [16]. All human MSC studies were approved by the Institutional Review Board of Hanyang University Hospital and were carried out in accordance with the approved guidelines; all participants provided written informed consents.

### 2.2. Lupus-Prone MRL.*Fas*^lpr^ Mice

Female MRL*.Fas*^lpr^ mice were purchased from the Jackson Laboratory (Bar Harbor, MA, USA). Mice were housed in specific pathogen-free conditions at 21–24°C and 40–60% relative humidity under a 12 h light/dark cycle. Female MRL*.Fas*^lpr^ mice were injected intravenously with PBS (vehicle, *n* = 6) or 4 × 10^5^ hMSCs/mouse (*n* = 6) once at the age of 12 weeks [14]. Survival rate and body weight were examined every week. Serum was collected every 3 weeks and stored at −70°C until use. The levels of anti-dsDNA IgG and total IgG in serum were measured by using ELISA kits purchased from Alpha Diagnostic International (San Antonio, TX, USA) and eBiosciences (San Diego, CA, USA), respectively, according to the manufacturers' instructions. All animal studies were approved by the Chungbuk National University Animal Experimentation Ethics Committee and were carried out in accordance with the approved guidelines.

### 2.3. RNA Interference

siRNAs targeting mouse chemokines were purchased from Bioneer (Daejeon, Korea). The following sequences were used: CCL2 (GenBank accession number NM 002982.3), 5′-CUC CGA AGA CUU GAA CAC UdTdT-3′, 5′-GAA ACA UCC AAU UCU CAA AdTdT-3′, 5′-GCU CGC GAG CUA UAG AAG AdTdT-3′; CXCL10 (GenBank accession number NM 001565.3), 5′-GGU CAC CAA AUC AGC UGC UdTdT-3′, 5′-GAG AUC AUU GCU ACA AUG AdTdT-3′, 5′-CAU GAA UCA AAC UGC CAU UdTdT-3′; CXCL12 (GenBank accession number NM 199168.4), 5′-GAU UCU UCG AAA GCC AUG UdTdT-3′, 5′-CCA GAG CCA ACG UCA AGC AdTdT-3′, 5′-CAA CAG ACA AGU GUG CAU UdTdT-3′. The following negative-control siRNA was used: 5′-CCU ACG CCA CCA AUU UCG UdTdT-3′. MSCs were transfected with 100 nM siRNAs using Lipofectamine RNAiMAX reagent (Thermo Fisher Scientific, Waltham, MA, USA) following the manufacturer's protocol. Cells were incubated at 37°C in a 5% CO_2_ incubator for 48 h [17].

### 2.4. Transwell Assay and Time-Lapse Imaging

In transwell assay, B cells (100 *μ*l) were added to the upper wells of transwell plates with a 5 *μ*m insert (Corning, Tewksbury, MA, USA). Various concentrations of chemokines or MSCs were added to the lower wells in 600 *μ*l of complete RPMI 1640 medium. The number of B cells that had migrated to the lower well over 1.5 h was counted using a flow cytometer [14].

For time-lapse imaging, MSCs (70 *μ*l of 0.3 × 10^6^ cells/ml) were seeded into the left chamber and B cells (70 *μ*l of 1 × 10^6^ cells/ml) into the right chamber of culture-insert *μ*-Dish^35mm^ culture dishes (ibidi GmbH, Martinsried, Germany). Time-lapse imaging was performed with a BioStation IM-Q microscope equipped with a 10x magnification objective (numeric aperture 0.5) in an environmental chamber kept at 37°C and 5% CO_2_ (Nikon Inc., Melville, NY, USA). Dishes were preincubated for 3 h in the chamber, and then, inserts were carefully removed. Images were acquired every 2 min for 12 h [18].

### 2.5. B and T Cell Functions

B cells were purified from spleen cells of MRL*.Fas*^lpr^ mice by a negative depletion method using a B cell isolation kit (Miltenyi Biotec, Auburn, CA, USA). T cells were purified using a T cell isolation kit (Miltenyi Biotec). Purity of both cell types was typically > 90%. To measure B cell proliferation, purified B cells (1 × 10^5^ cells/well) and MSCs (0.01–0.1 × 10^5^ cells/well) were mixed in 96-well plates. In some experiments, MSCs were cultured in the upper well and B cells in the lower well of transwell plates (Corning) to avoid cell–cell contact [14]. B cells were activated with lipopolysaccharide (LPS, 1 *μ*g/ml) on day 0 [19]. ^3^H-Thymidine (113 Ci/nmol; NEN, Boston, MA, USA) was added to the culture at a concentration of 1 *μ*Ci/well 54 h after LPS stimulation, and B cells were harvested using an automated cell harvester (Inotech, Dottikon, Switzerland) 72 h after LPS stimulation. The amount of ^3^H-thymidine incorporated into the cells was measured using a Wallac Microbeta scintillation counter (Wallac, Turku, Finland). The levels of IgM accumulated in culture medium for 72 h were determined by using an ELISA kit (Thermo Fisher Scientific). To measure T cell proliferation, concanavalin A (Con A, 1 *μ*g/ml) was used to specifically activate T cells [19]. The levels of T cell-derived IFN-*γ* were determined by using an ELISA kit (Bio-Techne, Minneapolis, MN, USA).

### 2.6. Western Blotting, RT-PCR, Quantitative PCR (qPCR), and ELISA

For western blotting, cell lysates were prepared as previously described [20]. Detergent-insoluble material was removed, and equal amounts of protein were fractionated by 10% SDS-PAGE and transferred to pure nitrocellulose membranes. Membranes were blocked with TBS/Tween 20 containing 5% bovine serum albumin for 1 h and then incubated with an appropriate dilution of primary antibody in the same buffer for 2 h. Blots were incubated with biotinylated secondary antibody for 1 h and then with HRP-conjugated streptavidin for 1 h. Signals were detected by enhanced chemiluminescence (Amersham Pharmacia Biotech, Piscataway, NJ, USA). Anti-mouse or anti-human antibodies against STAT1, phospho-STAT1, IDO, and GAPDH were purchased from Cell Signaling Technology (Danvers, MA, USA).

For RT-PCR, total RNA was isolated from MSCs using TRIZOL Reagent (Thermo Fisher Scientific). RNA was quantified using a spectrophotometer and stored at –80°C at a concentration of 1 mg/ml. cDNA was synthesized from 3 *μ*g total RNA using an RT kit (Bioneer). PCR was used to examine the expression levels of the following mRNAs: COX-2, TGF-*β*, IDO, CCL2, CCL3, CCL5, CXCL10, and CXCL12 [21]. PCR products were separated on 1% agarose gels and stained with 0.5 *μ*g/ml ethidium bromide [21].

Quantitative PCR was performed with a SYBR Green Master Mix (Elpis Biotech., Daejeon, Korea) in a StepOnePlus Real-Time PCR System (Applied Biosystems, Foster City, CA, USA). Relative mRNA levels in a sample were based on its threshold cycle in comparison with that of the housekeeping gene *β*-actin [22].

For ELISA, MSCs were cultured for 24 h, culture supernatants were collected, and the concentrations of PGE_2_, TGF-*β*, CCL2, CXCL10, and CXCL12 were measured by using ELISA kits according to the manufacturer's instructions (Bio-Techne).

### 2.7. Statistical Analysis

Data are presented as the mean ± SEM of at least three independent *in vitro* experiments performed in triplicates or six mice. To determine statistical significance, *p* values were calculated using one-way ANOVA (GraphPad Software, San Diego, CA, USA).

## 3. Results

### 3.1. hMSCs Ameliorate Lupus Progression in MRL.*Fas*^lpr^ Mice

**Figure 1:**
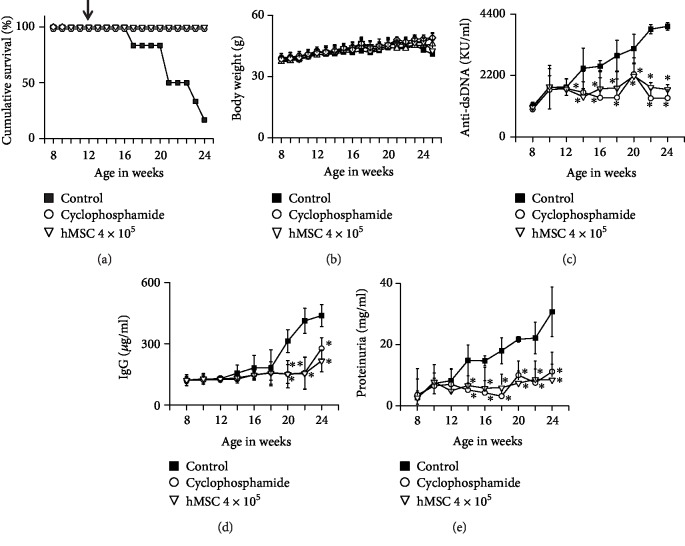
hMSCs ameliorate SLE development in MRL.*Fas*^lpr^ mice. MRL*.Fas^lpr^* mice were intravenously injected with vehicle (PBS, control; *n* = 6), hMSCs (4 × 10^5^ cells/mouse, *n* = 6), or cyclophosphamide (50 mg/kg, *n* = 6) once at the age of 12 weeks. Survival (a) and body weight (b) were measured every week. The levels of anti-dsDNA IgG (c) and total IgG (d) in serum and the level of proteinuria (e) were also measured. ^∗^*p* < 0.01 versus control.

We examined the therapeutic activity of hMSCs in MRL*.Fas*^lpr^ mice. hMSC-treated mice survived at least 24 weeks of age, which was much longer than the control mice (Figure 1(a)). hMSCs did not affect body weight (Figure 1(b)). The serum levels of anti-dsDNA (Figure 1(c)) and total IgG antibodies (Figure 1(d)) and proteinuria level (Figure 1(e)) were significantly lower in hMSC-treated mice than in control mice. As expected, the immunosuppressant, cyclophosphamide, used as a positive control ameliorated the disease (Figure 1). Overall, our data suggest that hMSCs ameliorate the progression of SLE in MRL*.Fas*^lpr^ mice.

### 3.2. Effect of hMSCs on Cytokine Gene Expression in the Kidney of MRL.*Fas*^lpr^ Mice

**Figure 2:**
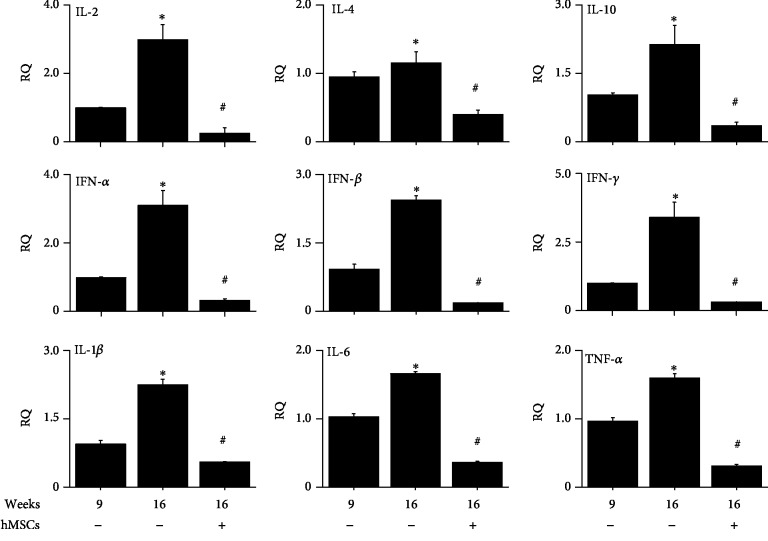
hMSCs decrease cytokine gene expression in the kidney of MRL.*Fas*^lpr^ mice. hMSCs (4 × 10^5^ cells/mouse, *n* = 5) were injected once at the age of 12 weeks. Total RNAs were isolated from the kidney at the age of 9 or 16 weeks (*n* = 5). The expression levels of cytokines were measured by qPCR. ^∗^*p* < 0.01 versus 9 weeks. ^#^*p* < 0.01 versus hMSC-untreated control (16 weeks).

We isolated total RNA from the kidney of MRL.*Fas*^lpr^ mice at 16 weeks of age (4 weeks after hMSC injection or vehicle injection). As additional control, we isolated total RNA from the kidney of MRL.*Fas*^lpr^ mice at 9 weeks of age (before hMSC injection). Compared to 9 weeks of age, the expression levels of all the cytokines examined increased at 16 weeks and were decreased by hMSC injection (Figure 2). These data suggest that hMSCs might inhibit the functions of inflammatory immune cells including T cells.

### 3.3. Effect of hMSCs on T and B Cells Isolated from MRL.*Fas*^lpr^ Mice

**Figure 3:**
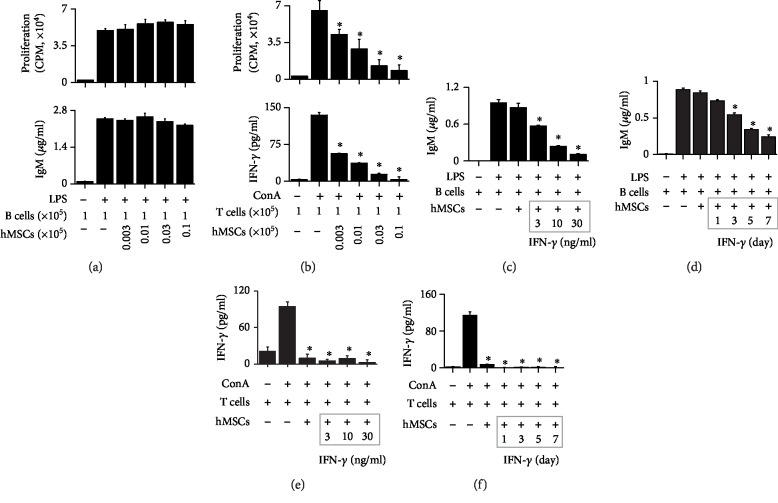
IFN-*γ* makes hMSCs capable of inhibiting MRL.*Fas*^lpr^ B cells. (a, b) hMSCs (0.003–0.1 × 10^5^ cells/well) and MRL*.Fas*^lpr^ mouse B (a) or T (b) cells (1 × 10^5^ cells/well) were cocultured for 72 h. B cells with lipopolysaccharide (LPS, 1 *μ*g/ml) and T cells were activated with concanavalin A (ConA, 1 *μ*g/ml). Proliferation of B and T cells was measured by the ^3^H-thymidine uptake assay (upper), and the levels of IgM (a) and IFN-*γ* (b) accumulated in culture medium were measured by ELISA (lower). (c–f) hMSCs were pretreated with IFN-*γ* (3–30 ng/ml) for 7 days (c, e) or with IFN-*γ* (10 ng/ml) for 1–7 days (d, f). hMSCs (0.1 × 10^5^ cells/well) and B cells (1 × 10^5^ cells/well) were cocultured for 72 h, and the levels of IgM were measured by ELISA (c, d). hMSCs (0.1 × 10^5^ cells/well) and T cells (1 × 10^5^ cells/well) were cocultured for 72 h, and the levels of IFN-*γ* accumulated in culture medium were measured by ELISA (e, f). ^∗^*p* < 0.01 versus control.

To examine whether hMSCs directly inhibit mouse T and B cells, we set up a xenogeneic assay system by coculturing hMSCs and immune cells isolated from MRL*.Fas*^lpr^ mice. hMSCs did not affect the proliferation of or IgM production by LPS-activated B cells (Figure 3(a)). However, hMSCs inhibited the proliferation of or IFN-*γ* production by ConA-activated T cells (Figure 3(b)). Nephritic kidney expresses high levels of inflammatory cytokines, including IFN-*γ*, which could improve the inhibitory capacity of MSCs [2]. Thus, we treated hMSCs with IFN-*γ* in dose- (Figures 3(c) and 3(e)) and time-dependent manners (Figures 3(d) and 3(f)). IFN-*γ*-activated hMSCs (IFN-hMSCs) inhibited the IgM production by LPS-activated mouse B cells (Figures 3(c) and 3(d)) and IFN-*γ* production by ConA-activated T cells (Figures 3(e) and 3(f)). These data suggest that naïve hMSCs inhibit only T cells and if they are activated by IFN-*γ*, they can inhibit both T and B cells.

### 3.4. IFN-hMSCs Inhibit Mouse B Cells in an IDO-Dependent Manner

**Figure 4:**
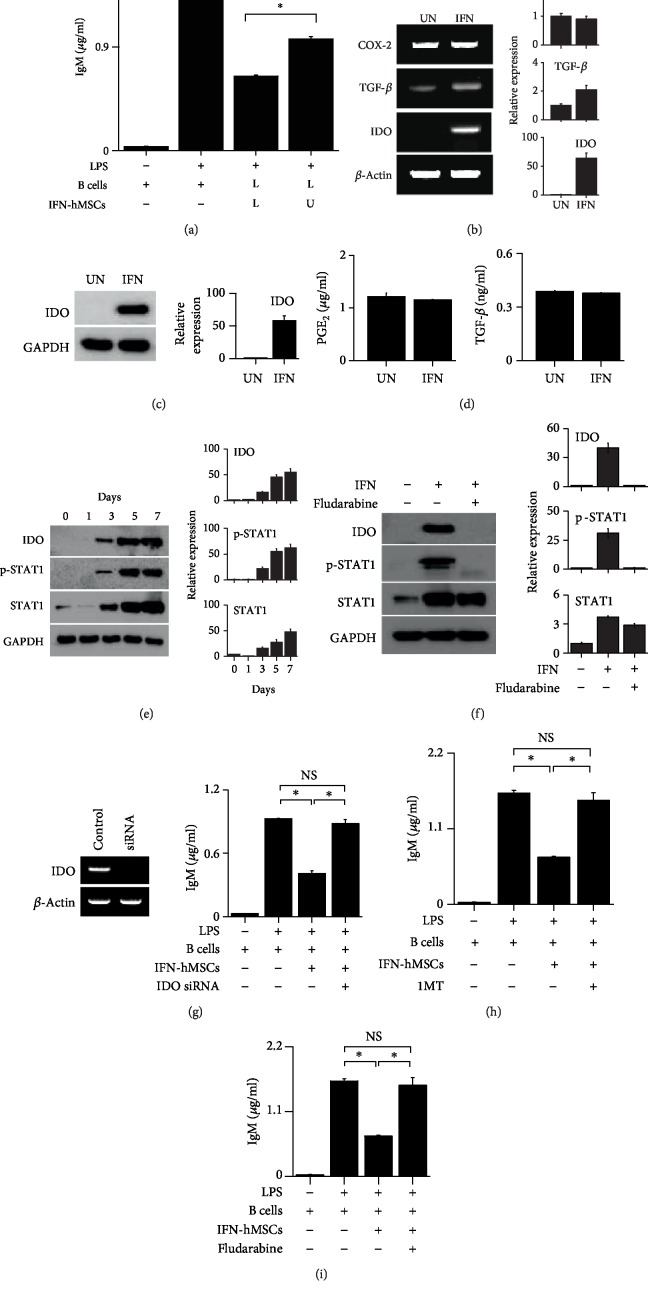
IFN-hMSCs inhibit MRL.*Fas*^lpr^ B cells in an indoleamine 2,3-dioxygenase-dependent manner. (a) IFN-*γ*-activated hMSCs (IFN-hMSCs; 1 × 10^4^ cells/well) were added to the upper (U) or lower (L) wells of transwell plates and MRL*.Fas*^lpr^ mouse B cells (1 × 10^5^ cells/well) to the L wells. After incubation with LPS for 72 h, the level of IgM accumulated in culture medium was measured by ELISA. (b) Total RNA was isolated from IFN-hMSCs, and the expression levels of COX-2, TGF-*β*, and IDO were analyzed by RT-PCR. (c) IDO protein level in IFN-hMSCs analyzed by western blotting. (d) Levels of PGE_2_ and TGF-*β* accumulated in IFN-hMSC culture medium over 24 h measured by ELISA (d). (e, f) hMSCs were treated with IFN-*γ* (10 ng/ml) for 7 days and the levels of IDO, STAT1, and p-STAT1 were analyzed by western blotting (e). In (f), the cells were treated with the STAT1 inhibitor fludarabine (10 nM) for 7 days. (g–i) IFN-hMSCs were transfected with IDO siRNA for 48 h (g) or treated with the IDO inhibitor 1-methyltryptophan (1MT, 100 *μ*M) for 7 days (h) or fludarabine (10 nM) for 7 days (i). The level of IgM accumulated in culture medium was measured by ELISA. ^∗^*p* < 0.01 (*n* = 3). NS: not significant. (b, c, e, and f) Band areas were analyzed by ImageJ software (NIH, Bethesda, MD, USA), and data were presented as ratios versus UN (b, c), day 0 (e), and IFN/fludarabine-untreated group (f) (*n* = 3).

Much has been learned about the mechanisms of T cell inhibition by MSCs, whereas little is known about the effects of MSCs on B cells. Thus, we examined the effect of IFN-hMSCs on mouse B cells in greater detail. To assess whether IFN-hMSCs inhibit mouse B cells in a soluble factor- or contact-dependent manner, we used a transwell assay. When we added IFN-hMSCs and mouse B cells to the lower wells, thereby allowing cell–cell contact, IFN-hMSCs strongly inhibited IgM production by B cells (Figure 4(a)). When we added IFN-hMSCs to the upper wells and mouse B cells to the lower wells, thereby preventing direct cell–cell contact, the inhibition was weaker (Figure 4(a)). These data imply that IFN-hMSCs inhibit mouse B cell functions in both contact- and soluble factor-dependent manners. Naïve hMSCs expressed the immunosuppressive soluble factors cyclooxygenase-2 (COX-2), PGE_2_, and TGF-*β*, and IFN-hMSCs additionally expressed IDO (Figures 4(b)–4(d)), suggesting that IFN-hMSCs might use IDO for B cell inhibition. IFN-*γ* increased IDO expression by phosphorylating STAT1 in a time-dependent manner (Figure 4(e)); STAT1 inhibitor fludarabine inhibited STAT1 phosphorylation and IDO expression (Figure 4(f)). The ability of IFN-hMSCs to inhibit B cells was abolished by the transfection of IFN-hMSCs with IDO siRNA (Figure 4(g)) and treatment with IDO inhibitor 1MT (Figure 4(h)), or fludarabine (Figure 4(i)). These data suggest that IFN-hMSCs inhibit mouse B cell functions in an IDO-dependent manner.

### 3.5. IFN-hMSCs Inhibit Mouse B Cells in a CXCL10-Dependent Manner

**Figure 5:**
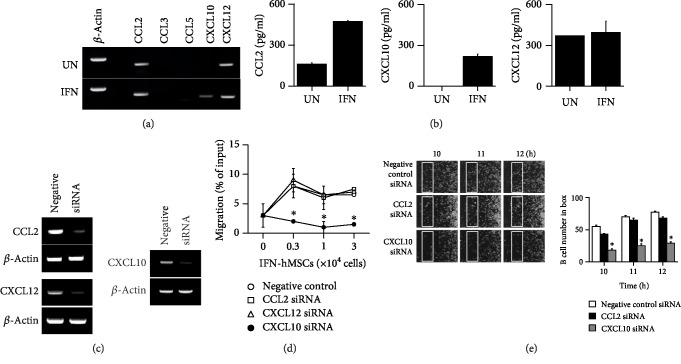
IFN-hMSCs inhibit MRL.*Fas*^lpr^ B cells in a CXCL10-dependent manner. (a, b) Expression levels of chemokines in MSCs measured by RT-PCR (a) and ELISA (b). UN: untreated hMSCs. IFN: IFN-treated hMSCs. (c–e) IFN-*γ*-activated hMSCs (IFN-hMSCs) were transfected with negative-control, CCL2, CXCL10, or CXCL12 siRNA. Expression levels analyzed by RT-PCR (c). IFN-hMSCs (0.3‐3 × 10^4^ cells/well) were added to the lower wells and MRL*.Fas*^lpr^ mouse B cells (1 × 10^5^ cells/well) to the upper wells of transwell plates with a 5 *μ*m insert. After 1.5 h, the number of B cells migrating to the lower well was determined (d). For time-lapse imaging, IFN-hMSCs (70 *μ*l of 0.3 × 10^6^ cells/ml) were seeded into the left chamber and B cells (70 *μ*l of 1 × 10^6^ cells/ml) into the right chamber of culture-insert *μ*-Dish^35mm^ culture dishes. Images were acquired every 2 min for 12 h (*n* = 3). Representative photos are shown. The number of B cells passing through the white boxes is shown on the right (e). ^∗^*p* < 0.01.

Next, we examined how IFN-hMSCs contact mouse B cells. IFN-hMSCs adhere to dishes and mouse B cells grow in suspension, suggesting that B cells might migrate toward IFN-hMSCs after sensing IFN-hMSC-derived chemokines. Thus, we assessed the profiles of chemokines expressed by IFN-hMSCs and chemokine receptors in mouse B cells. Naïve hMSCs expressed CCL2 and CXCL12, and IFN-hMSCs additionally expressed CXCL10, suggesting a potential role of CXCL10 in MSC–B cell interactions (Figures 5(a) and 5(b)). To prove this hypothesis, we knocked down CCL2, CXCL10, or CXCL12 with respective siRNAs (Figure 5(c)). IFN-hMSCs transfected with CCL2 or CXCL12 siRNA, but not IFN-hMSCs transfected with CXCL10 siRNA, induced mouse B cell migration in transwell assay (Figure 5(d)). We performed time-lapse imaging to assess mouse B cell migration towards IFN-hMSCs at the single-cell level. We placed IFN-hMSCs on the left side of an imaging chamber and mouse B cells on the right side and acquired images every 2 min for 12 h (Supplementary [Supplementary-material supplementary-material-1]). Representative images and the number of mouse B cells passing through a selected area (white box) are shown in Figure 5(e). IFN-hMSCs transfected with negative-control or CCL2 siRNA induced B cell migration, whereas CXCL10 siRNA-transfected IFN-hMSCs did not (Figure 5(e)). Overall, these data suggest that IFN-hMSCs attract mouse B cells for contact-dependent inhibition in a CXCL10-dependent manner.

## 4. Discussion

MRL.*Fas*^lpr^ mice have been widely used to evaluate the preclinical efficacy of hMSCs against SLE. These mice lack the *Fas* gene and spontaneously develop SLE-like symptoms, including an increase in anti-dsDNA antibodies in blood, and develop severe nephritis [23]. Similar to SLE patients, the symptom severity in MRL*.Fas*^lpr^ mice depends on gender: female mice die at an average age of 17 weeks and males at 22 weeks. Several preclinical studies have demonstrated that hMSCs ameliorate SLE progression in MRL*.Fas*^lpr^ mice. Lee et al. [14] showed that the transfer of hMSCs increased MRL.*Fas*^lpr^ mouse survival, decreased T cell infiltration in the kidneys, and reduced T cell cytokine expression. Lee et al. [24] also showed that hMSCs in combination with prednisone or mycophenolate mofetil had a better therapeutic effect than single therapy in MRL.*Fas*^lpr^ mice; the following readouts were used in this study: prolongation of survival, decrease in anti-dsDNA and total IgG levels in serum, decrease in cytokine gene expression in spleen cells, and decrease in inflammatory cell infiltration into the kidney. Liu et al. [25] showed that hMSCs reduced proteinuria level, serum anti-dsDNA antibody level, and renal damage in MRL.*Fas*^lpr^ mice. In agreement with these previous data, our data confirm that xenogeneic hMSCs ameliorate SLE progression in MRL.*Fas*^lpr^ mice. hMSC-injected mice showed decreased antibody production and lowered cytokine expression, which suggested the inhibition of immune cells including T and B cells. Overall, the preclinical studies cited above and our present study suggest that hMSCs efficiently ameliorate lupus progression in MRL.*Fas*^lpr^ mice.

Although human or mouse MSCs have been reported to inhibit species-matched T and B cells [26–28], it has been unclear whether hMSCs would directly affect the functions of xenogeneic T and B cells in MRL.*Fas*^lpr^ mice. We proved here that naïve hMSCs inhibited mouse T cells but not mouse B cells. However, hMSCs might be able to inhibit B cells in mice as the disease progresses. The previous studies [26–28] and our data demonstrate that the nephritic kidney of MRL.*Fas*^lpr^ mice expresses high levels of many inflammatory cytokines, including IFN-*γ*. IFN-*γ* upregulates the expression of IDO by hMSCs [2, 29, 30]. IDO is a powerful immunosuppressive enzyme catalyzing the first step in L-tryptophan catabolism [31]. IDO depletes L-tryptophan and produces kynurenines, which might inhibit B and T cells [31]. IFN-hMSCs inhibit the proliferation of T and B cells and IgM production by B cells in an IDO-dependent manner [29, 30]. Our data confirm that IFN-hMSCs inhibit mouse B cell functions in an IDO-dependent manner. Here, we also show that IFN-*γ* increases CXCL10 production by hMSCs. Although IFN-hMSCs produced several chemokines, including CCL2, CXCL10, and CXCL12, they used only CXCL10 to attract mouse B cells. As we previously reported, hMSCs use CCL2 to attract mouse T cells [14]. Together, these data suggest that hMSCs attract mouse T and B cells for contact inhibition by using different chemokines. Overall, it is well documented that MSCs inhibit T cell functions in both contact- and soluble factor-dependent manners [14]. Our present study demonstrates that IFN-hMSCs inhibit B cells in the same way.

## 5. Conclusion

Our study has an inevitable limitation: our data might not be clinically meaningful, since we used hMSCs as effector cells and mouse T and B cells as target cells. However, our data provide important preclinical information on how hMSCs ameliorate SLE progression in lupus-prone MRL.*Fas*^lpr^ mice. At an early stage after injection into mice, naïve hMSCs inhibit mouse T cells, but not mouse B cells. Later, if hMSCs are activated by IFN-*γ* in mice, they can inhibit mouse B cells in a CXCL10- and IDO-dependent manner. Our data might help to understand how hMSCs ameliorate SLE progression in MRL.*Fas*^lpr^ mice, which are widely used to evaluate the preclinical efficacy of hMSCs against SLE.

## Data Availability

The data used to support the findings of this study are included within the article.

## Supplementary Materials

Supplementary Materials Supplementary Video 1: migration of mouse B cells to IFN-hMSCs. IFN-hMSCs transfected with negative control siRNA (a) and CXCL10 siRNA (b) were loaded in the left chamber. Mouse B cells were loaded in the right chamber. B cell migration was imaged every 2 min for 12 h. Related to Figure 5(e).
